# Camels and Climate Resilience: Adaptation in Northern Kenya

**DOI:** 10.1007/s10745-016-9858-1

**Published:** 2016-11-12

**Authors:** Elizabeth E. Watson, Hassan H. Kochore, Bulle Hallo Dabasso

**Affiliations:** 1 Department of Geography, University of Cambridge, Downing Place, Cambridge, CB2 3EN UK; 2 Max Planck Institute for Social Anthropology, Advokatenweg 36, 06114 Halle (Saale), Germany; 3 Kenya Agricultural and Livestock Research Organisation, P.O. Box 147, Marsabit, Kenya

**Keywords:** Camels, Climate change, Adaptation, Resilience, Pastoralism, Kenya

## Abstract

In the drylands of Africa, pastoralists have been facing new challenges, including those related to environmental shocks and stresses. In northern Kenya, under conditions of reduced rainfall and more frequent droughts, one response has been for pastoralists to focus increasingly on camel herding. Camels have started to be kept at higher altitudes and by people who rarely kept camels before. The development has been understood as a climate change adaptation strategy and as a means to improve climate resilience. Since 2003, development organizations have started to further the trend by distributing camels in the region. Up to now, little has been known about the nature of, reasons for, or ramifications of the increased reliance on camels. The paper addresses these questions and concludes that camels improve resilience in this dryland region, but only under certain climate change scenarios, and only for some groups.

## Introduction

In northern Kenya, the role played by camels in livelihoods has increased. Pastoralists who historically depended on cattle have started to herd the one-humped camel (*Camelus dromedarius)*. The shift has been seen as an adaptation to climate change, ‘a means to build climate resilience’ (Toulmin 2009), as camels can survive severe droughts and continue to contribute to household nutrition and economy in dry periods (Dahl and Hjort 1976; Wilson *et al.* 1990; Hülsebusch and Kaufmann 2002). It appears to be a success story, a locally driven initiative by people who are typically thought to be amongst the most vulnerable to climate change and to have ‘low adaptive capacity’ (Boko *et al*. 2007). The initiative has also been taken up by development organizations, which have started to distribute camels that they view as ‘the most resilient livestock.’^1^ To date, little research has been carried out into the nature of this development, and our aim is to examine the processes by which a drought-resistant animal (camel) is being increasingly adopted by pastoralists, and the extent to which it fulfils claims of being a form of adaptation that improves resilience.

Adaptation to climate change is accepted as urgent and necessary. The strong imperative to identify an appropriate ‘adaptation pathway’^2^ belies the complexity of defining what adaptation is, who should do it, whom it should be for, and what that adaptation should be to (Adger *et al.* 2009b; Conway 2009; Pelling 2011). Adaptation operates in the context of multiple uncertainties and unknowns: we don’t know exactly what the impact of global climate change at the local level is or will be, or how stresses associated with it will intersect with other dynamic processes operating across multiple scales (Vincent 2007). Amidst this complexity, localised case studies have a lot to offer through their examination of ‘the lived experiences of resource-dependent societies in the developing world in coping with climate variability’ (Adger *et al.* 2003). We explore processes taking place on Marsabit Mountain in northern Kenya. The case is not the first wave of interest in camels in the region, however: increasing numbers of camels were reported for Samburu in the 1980s (Sperling 1987), and for Pokot in the 1950s and 1960s (Bollig 1992). These historic cases provide some insights into the advantages and disadvantages of keeping camels, but the current case is different in that it is a response to opportunities and challenges that have emerged since the mid-1990s, and the way it is being heralded by policy-makers as an adaptation to climate change and a form of resilience is also new.

Although agriculture in general is appreciated to be one of the most important sectors for livelihoods and human security in Africa (Boko *et al.* 2007), and despite attention to the importance of drought-resistant crops (The Royal Society 2009), the use of drought-resistant livestock as a climate adaptation strategy has received scant attention. Studies by Thornton *et al.* (2009) and by Toni and Holanda (2008) mention their importance given future climate change scenarios, but up to now few, if any, have examined their adoption as ‘a means to build climate resilience’ in practice. There is literature on herd diversification as a regular strategy used by pastoralists to spread risk, but this does not cover innovative forms of diversification to drought-resistant livestock (Dahl and Hjort 1976). A further literature discusses diversification to non-pastoral livelihoods as a response to new opportunities and new pressures since the 1970s (Little *et al.* 2001a; McCabe *et al.* 2010), but again, there is little discussion of the new forms of pastoral diversification. We here address these issues.

## Pastoralism, Adaptation and Resilience

Pastoralists are understood here as those who derive (or who aspire to derive) some or all of their livelihoods from livestock (Little *et al.* 2001a; Adano and Witsenburg 2008). Climate change adaptation is seen as particularly important for pastoralists because of their high dependence on rain-fed natural resources. In debates about pastoralists and climate change adaptation, two key positions are evident (see also Robinson and Berkes 2010). On the one hand, many contend that climate change will push already-stressed pastoralist systems into a state of collapse, leading to a vicious circle of poverty, environmental degradation and violence (see Catley *et al.* 2013 for review). On the other hand, other researchers argue that pastoralism has inherent flexibility and resilience: under future climate change scenarios it may have ‘an important role to play where other livelihoods are likely to fail’ (Nassef *et al.* 2009). In practice, there is widespread appreciation that the situation is more complex, but polarized views that either support pastoralism or try to find alternatives to it are still influential. By exploring the nature and reasons for practices on the ground, we contribute to a more nuanced account of pastoralist livelihoods and related climate change adaptation and policy.

As we examine the question of whether camels improve climate resilience of pastoralist communities, the paper is also situated within debates about resilience. The concept of resilience, it has been argued, provides a useful way of thinking through some of the complexities of adaptation (Nelson *et al.* 2007). Broadly conceived, resilience is understood as the ability to cope with the various and sometimes unexpected shocks and stresses of climate change, and as being at ‘the core of adaptation’ (Conway 2009). This approach is termed here the ‘literal’ approach as it builds on commonplace understandings of resilience that refer to a ‘quality or fact of being able to recover quickly or easily from, or resist being affected by, a misfortune, shock, illness, etc.’^3^ In relation to contemporary climate change policy, such literal understandings of resilience are a function of development: ‘if people are better fed and in better health, and have better access to education, jobs and markets, then they will be more resilient to climate change’ (Conway 2009).

Scholars have also developed more specialised ‘resilience framework’ approaches, in which resilience is understood as ‘the amount of change a system can undergo and still retain the same function and structure while maintaining options to develop’ (Nelson *et al.* 2007). Derived from the field of ecological modelling, this approach emphasises non-linear dynamics, surprises and uncertainty. Its characteristics suggest that it is highly suited to dryland pastoralist systems as they are characterised by non-equilibrium dynamics (McCabe 2004; Robinson and Berkes 2010). A similar analysis is not presented here, however, as we concluded that the application of the resilience framework to the present case did not produce new insights. As others have found (Cannon and Müller-Mahn 2010; Hornborg 2013; Bollig 2014), instead it tended to occlude uneven power relations that emerged as critical. For reasons of space these ‘non-findings’ cannot be presented here, and the current paper limits itself to an analysis of resilience in its literal sense.

## The Study Context

Marsabit County is the largest County in Kenya, and has a population of 291,166.^4^ Endemic poverty is high, with 91.7 % of the population living below Kenya’s poverty line^5^; there is limited access to government services and food insecurity is commonplace. Predictions of the impact of global climate change are uncertain, but the consensus is that the area is likely to ‘face more climatic variation and extreme weather events’ (Conway 2009), including becoming warmer, drier, and more likely to experience episodes of drought, as well as episodes of heavy rain and flooding (Boko *et al.* 2007; Dabasso and Okomoli 2015).

**Fig. 1:**
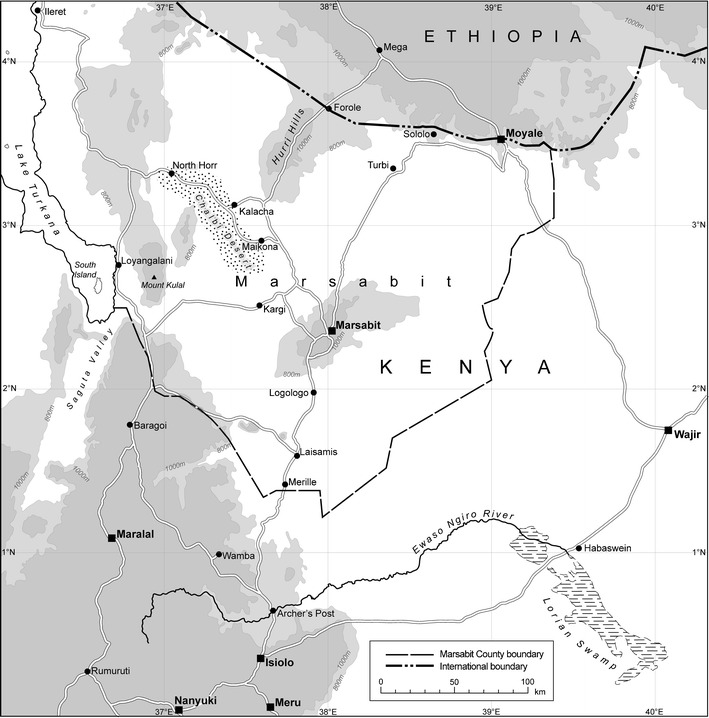
Northern Kenya

The majority of land in the region is below 800 m.a.s.l., where rainfall is low (between 200 and 250 mm per annum), uneven and unpredictable (Adano and Witsenburg 2008). Breaking the monotony of the rocky dryland plains are isolated but significant regions of higher land, such as those around Marsabit Mountain, the Hurri Hills, and Mount Kulal (Fig. 1). These regions have higher rainfall: Marsabit weather station registers averages between 600 and 800 mm per year. The region is multi-ethnic, with Borana, Gabra, Rendille and Samburu being the key groups for this study. Somali groups such as the Adjuran, Degodia and Garre are found to the north-east and east of the area under discussion. All of these groups have historically depended on livestock for most or all of their livelihoods, and have practised varying degrees of mobile pastoralism (O’Leary 1994). The Gabra, Rendille and Somali groups historically preferred camels, the Borana and Samburu, cattle (all also keep small stock). While cattle, sheep and goats have a very long presence in the region (c. 5000 years), there is some debate about when exactly camels arrived (some archaeologists suggest first millennium BC^6^; others only AD 1300–1600^7^). In oral traditions, specialized cattle and camel herding dates back to at least the sixteenth century and has been intertwined with conflicts and inter-group alliances (Schlee 1989).

Since the 1950s and 1960s, the livelihoods of mobile pastoralists have been profoundly transformed by both sedentarization and diversification. In the 1950s, the British encouraged cultivation on Marsabit Mountain; in the 1960s and 1970s, missionaries resettled pastoralists who were destitute following droughts or conflict, and encouraged farming (Fratkin and Roth 2005). In the decades that followed, pastoralists settled around towns and water points, attracted by access to food relief, services and town life, or ‘pushed’ by an inability to practise mobile pastoralism following livestock loss through drought, conflict or disease (ibid.). Little *et al.* (2001a) argued that diversification out of pastoralism has been pursued by relatively wealthy herders who chose to make use of new opportunities, and by poorer herders who were ‘pushed’ to diversify. Middle-wealth pastoralists were less likely to diversify, and, additionally, those who continued to practise mobile pastoralism exhibited lower levels of poverty (Little *et al.* 2008). Diversification away from pastoralism in this region, they concluded, tended ‘to generate low incomes and thus may actually increase risk during periods of stress’ (Little *et al.* 2001a).

In 2012, many of the people discussed in this paper were living in settlements, but livestock keeping was still important to their livelihoods and their identity (see also Adano and Witsenburg 2008). Households continued to split their animals into different herds for grazing by species (small stock, cattle and camels) and into a home camp and a more mobile, satellite camp. Patterns of mobility and herd splitting depended on herd composition, on weather, and on grass and labour available, as well as on personal inclination (Dahl and Hjort 1976; O’Leary 1994; Cordaid 2012). Sedentary lives and mobile livestock keeping practices were often combined in different and changing ways.

Historically, cattle and camels were kept in different ecological zones. Camels thrived in the hot dry lowlands, and cattle on the higher moister ground. Schlee (1989) explained, ‘[t]hese highlands were too cold and wet for camels… [if kept there] they would start to cough and lose weight.’ In 1998 and 2000 Adano and Witsenburg researched pastoral sedentarization around Marsabit, and observed that, unlike cattle, ‘camel ownership is quite low on the mountain and furthermore camels rarely stay at residential homes on the mountain’ (2008). Subsequently, the spatial distribution of different livestock species has changed. In 2012, camels were observed browsing and being kept on the mountain.

The shift from cattle to camels has been profound on various fronts: first, ecologically, it represents a very different use of the environment. Camels ‘feed on plants or parts of plants not eaten by more conventional livestock’ (Wilson *et al.* 1990), such as dwarf shrubs and browse. Second, economically, livelihoods that depended on managing, rearing, selling and consuming cattle products have come to rely on camels. Third, culturally, many people who were taking up camels are Borana, who historically had cattle at the centre of their social and ritual worlds. It was often said that ‘to be Borana is to have cattle.’ One elderly man explained, ‘the Borana think their cattle is their *gutu* [a ritually-significant length of hair grown by Borana men]. If you have no cattle, you have no *gutu.’* 8 An elderly Borana woman described cattle as the Borana’s ‘umbilical cord,’ highlighting a further sense of bodily connection.^9^ In the past, some Borana owned camels, but these were a minority (interviews, see also Oba 2013). Even in 2012, it was considered taboo for certain Borana clans to drink camel milk, eat camel meat or say the word ‘camel,’ instead using the phrase ‘that long-necked thing.’^10^ Fourth, politically, according to Schlee, cordial and mutually supportive relations between neighbouring ethnic groups such as the Borana and the Gabra were possible in the past because ‘between the Gabbra (sic) and Borana, there was little grazing competition… because at least the majority of the former specialized in camels and the latter in cattle’ (1989).^11^ A Borana move into camel keeping would disrupt this spatial specialization, and new competition might lead to conflict. In sum, while at first glance the question ‘do camels deliver climate resilience’ appears straightforward and primarily ecological, on closer inspection it is complex and related to multiple fundamental dimensions of life.

## Methods

Marsabit Mountain was chosen for this case study as a result of a combination of ‘purpose and serendipity’ (Stratford and Bradshaw 2016). The authors had been carrying out research in this region on livelihoods, culture, development and environment since 1999.^12^ During fieldwork in 2010, on a journey back to Marsabit town from the lowlands, they were struck by the astonished comments of Gabra fellow passengers at the sight of Borana taking camels along the road through town to water: ‘How can that be?’ they asked; ‘how do they know how to manage them?’ Their questions suggested that there were new camel-based livelihoods on the mountain, and, in September/October 2012, the authors spent four weeks examining the extent and nature of these new camel-keeping practices. The first questions that drove the methods employed included: In what ways are these practices ‘new’? Are they linked to particular areas on the mountain? How do they relate to use of land, water and other resources? What inputs do the practices require, and what are their benefits and challenges?

The settlements and households in the area under study were quite widely distributed. In order to examine variation in camel-keeping practices across locations on the higher land, we made a circuit of the mountain (a distance of approximately 150 km) and carried out interviews in all the settled areas (defined as settlements above 800 m.a.s.l.). In addition, visits were made to lowland areas to compare camel-keeping practices on higher land with the longer-held lowland practices. Semi-structured interviews were the main methodology, as they allowed us to gain the trust of the interviewee and to obtain more in-depth information on history, motivations, forms of knowledge and management, experiences, successes and difficulties. A questionnaire had been designed for use, but in the early phases of the research we found that the information it generated was inaccurate and interviewees appeared uncomfortable with answering questions, particularly on herd sizes.^13^ As extended periods were spent with particular households, some of whom were visited on more than one occasion, it was found that initial estimates of numbers of livestock often varied quite extensively from the number that came home in the evening to the livestock enclosure, or were related at later points in an interview. The semi-structured interviews employed a combination of open and closed questions. The open questions were pursued through a narrative approach that encouraged the interviewee to ‘tell the story’ of his/her family, of how he/she had obtained camels, or had managed to cope with recent challenges, such as drought events. This narrative approach generated rich material that placed the contemporary changes in context and enabled the interviewee to discuss processes more on their own terms. Closed questions were also included in the interview, and a checklist of questions was used that was derived from the questionnaire, in order to ensure certain information was collected when it had not been generated through the narrative approach. These included questions such as (in summary): Do you have camels? When/where did you get your first camel? What kind(s) is/are your camels? What other livelihoods do you practice? What did you do in the last (2011) drought? Have any of your camels died prematurely (why/how)? Further questions explored medicines and knowledge about treating camels, reproduction, milk sales, household economy, and matters related to culture such as bridewealth. Questions also compared cattle and camel pastoralism.

Interviewees were selected through a combination of purposive and snowball sampling (Bernard 2011). As we travelled around the settlements on the mountain we sought out people who were keeping camels by drawing on existing contacts and experience. Those interviewed were also asked who else had obtained camels and they were also interviewed. Camel herders in the lowlands who had not switched to camels were interviewed for comparison. We took care to include in our non-probabilistic interview sample a range of people: older and younger, male and female, richer and poorer, as well as members of different ethnic groups. We also interviewed key informants such as livestock professionals, veterinarians, and elders (male and female) who were experts in cultural matters (ibid.). In total, in addition to multiple informal conversations, 53 formal semi-structured interviews were carried out with 27 with camel-herding households (20 on the mountain, 7 in the lowlands), 26 others including cattle herders, borehole managers, knowledgeable elders, traders, and livestock professionals. Although some very basic quantitative data are presented, these figures remain illustrative and are not representative (Stratford and Bradshaw 2016). The study is largely qualitative and reflects the interviewees’ subjective understandings of camel-related developments as we understood and interpreted them. Although it is not representative, the case provides a reasonable but preliminary account of how some people are responding to challenges and the extent to which the shift to camels is a climate adaptation that improves resilience.

We carried out interviews in local languages, and translation into English was in situ. In most interviews, detailed notes were taken by hand and typed up later. Some interviews were recorded (with permission) and were transcribed by the first author. The data have been subject to narrative analysis focusing on how processes developed over time and how they have been understood and experienced. They have also been subject to thematic analysis exploring how camel-keeping practices varied among people of different ethnic groups, in the highlands and lowlands, across livelihood and wealth groups, and between different genders. All quotes from local people are cited using pseudonyms.

We first place the changes in their historical context and identify the key actors in the process. Second, we examine the reasons for the changes to explore the extent to which they represent adaptation to climate change or to other developments; we also present material beginning the exploration of the extent to which camels help to build resilience. Third, our discussion of the social, cultural and political dimensions of the changes provides a more rounded multi-dimensional understanding of the resilience related to camels. Fourth, a discussion of the risks entailed and nature of participation in the new camel economy explores the question of whether or not and for whom camels provide improved resilience. In these four substantive sections, findings and discussion are woven together. The results are summarized in the conclusion.

## The History of New Camel-Keeping Practices

It is not unreasonable to assume that camels have been increasingly adopted as a result of the number of development organizations working in this region. In colonial times, Marsabit County was part of the Northern Frontier District. Colonial development policies were largely limited to the establishment of grazing areas for different ethnic groups, the sinking of boreholes, and the encouragement of agriculture on the mountain (Schlee and Shongolo 2012; Oba 2013). From the 1960s, Christian missions became important actors, but in practice, the majority of their development activities focused on providing food relief to destitute pastoralists, and encouraging diversification of livelihoods away from pastoralism (Fratkin and Roth 2005).

From 1976 to 1985, Marsabit was the centre of a large UNESCO Man and Biosphere programme, known as the Integrated Project in Arid Lands (IPAL). The programme focused on research and policy to improve human use of the environment (Fratkin 1991; Lamprey and Yusuf 1981). As Fratkin’s (1991) history has shown, much of the programme’s work was shaped by ideas linked to Hardin’s (1968) ‘tragedy of the commons’ and focused on the inefficiencies and environmental degradation of pastoralist systems. Research has argued that its focus on market-oriented pastoralism failed to translate into meaningful support for the majority of herders (Fratkin 1991).

Historically, therefore, practical support for pastoralism in general, and for camels in particular, was thin on the ground. One exception was a ‘Mobile Outreach Service’ (MOS) that involved a team of development workers travelling by camel to take development to ‘the most remote, least hospitable and least secure parts of the country’ (Field 2005). This project was funded by Farm Africa and emerged from the experiences of IPAL.^14^ The MOS project was not mentioned by interviewees^15^ but several did mention a small off-shoot initiative: a ‘camels for school milk’ project, also funded by Farm Africa. Starting in 1991, this project provided camels to 30 schools across Samburu, Marsabit and Moyale Districts (Field 2005). Farm Africa has long gone from the region but, more than two decades later, some new camel herders mentioned their project as an example that had influenced their decision to switch to camels.

The MOS notwithstanding, most interviewees regarded the new interest in camels as an initiative of the people themselves. For example, Halake Elema, a knowledgeable Borana elder, explained:

It happened in two ways: there are those people who always used to have camels but they kept them with the Gabra. Some of these people started to ask for their camels, and they brought them here [to the mountain]. The others, they started selling cattle and buying camels. This happened roughly 16 years ago [1996].^16^

The elder’s view was partly borne out by our data. Of 27 camel-owning households, 20 represented cases where the camels were being kept on the mountain.^17^ These can be divided into three groups. The first comprised only two Borana households who said they had camels ‘from long ago,’ and had inherited camels from their parents. One of these households used to live with Gabra, herding their camels together in lowlands but, following conflict between Borana and Gabra in the 1990s, they moved with their camels to the mountain.

The second group (*n* = 9) fitted the elder’s description of those who ‘started selling cattle and buying camels.’ The dates when these households first purchased camels ranged from 1996 to 2009, and they tended to have herds of around ten camels. Two had fewer, and one had many more (42). The camels had mostly been purchased in Moyale and Wajir markets, and they were ‘Somali’ camels, a larger and more productive breed than the small and hardy camels of the lowland Gabra and Rendille (Hülsebusch and Kaufmann 2002).^18^

The third group (*n* = 9), not referred to by our interviewee, included those who received a camel from one of three different organizations involved in distributing camels between 2003 and 2012 as part of a livelihood support programme. The first was the government Arid Lands Resource Management Project funded by the World Bank. According to the project manager, the decision to distribute camels followed requests by communities during participatory development planning meetings.^19^ Between 2003 and 2010, they distributed 200 camels to six communities on the mountain (each received 30–45). The camels were distributed through Community Development Committees who selected beneficiaries, each of whom contributed 30 % to the cost of the camel (approximately 7000 Shillings [c. £50]^20^). Although their distributions finished by 2010, the project manager commented that, ‘now all the communities are asking for camels.’^21^ The second organization was the NGO Pastoralist Integrated Support Programme (PISP), which distributed approximately 300 camels on the mountain during a similar period, under the same arrangements with communities. The third organization was the Pastoralist Community Initiative and Development Assistance (PACIDA) that in 2012 carried out the largest distribution: 2075 camels were provided mostly to communities living in the lowlands. In this case, each recipient gave two or three sheep or goats in exchange for a camel.^22^ The recipients we interviewed did not distinguish between these different organizations, and referred to all as ‘project.’ All the camels distributed were also of the ‘Somali’ type. Of the nine recipients interviewed in 2012, four still had only one camel; four had two camels as the ‘project’ camel they had received had given birth once; and one man had three camels, as the camel that he had received in 2009 had given birth twice.

This history reveals that camels started to be kept on the mountain in the mid-1990s, following worsening conflict between Borana and Gabra, and as some people started to sell cattle and buy camels. It was only later that development organizations started to contribute to the increasing number of camels. The number of camels on the mountain held by our interviewees that had been distributed by organizations is also much smaller than those that had been bought. It is possible to conclude, therefore, that it is the pastoralists – not development organizations - who have been responsible for this new direction in livestock keeping.

It is difficult to obtain an accurate figure of the number of camels on the mountain overall. The 2009 Census of Livestock Population counted 915 camels in Marsabit (which relates to the area around the mountain), but in 2012 a Government Veterinary Officer estimated the number on the mountain to be around 6000.^23^ Although our data are insufficient to quantify the increase in camels, our observations and interviews suggest that the increase has been more than just idiosyncratic. As the same Veterinary Officer commented: ‘people are opting for camels because of more desert … Since the climate has changed over the past four-to-five years, and the drought has become constant.’ His words also lead to further considerations of the reasons why herders have decided to make such a change.

## Reasons for Change

In interviews, all related the increased preference for camels to increased experiences of drought. As Halake Elema continued:

The reason the Borana started keeping camels is because the *ola* (drought) became terrible. The pasture was not growing and crops were also not doing well. So people thought, the camel seems to be surviving, so let’s try the camel.^24^

Others quoted the Borana saying ‘*ooanti gaala seessa looni lubbu*’, (‘the hardship which is a minor thing to the camels is dangerous for the cattle’),^25^ and added comments like ‘the camel can’t hear drought’,^26^ ‘the camel is like a strong man, who can stay without water for some days’^27^ or ‘the camel prays for drought’.^28^

Local perceptions of increasing drought and overall reduced rainfall are consistent with existing analysis indicating a decline in annual rainfall over a 50-year period (1961–2010) (Dabasso and Okomoli 2015). But the camel’s ability to withstand drought was only one reason it was considered an attractive prospect. The camel’s high and rising economic value was equally celebrated:

Camels have more benefits than the animals with hooves… The first benefit is that they can withstand drought. The second is that they can fetch good money. A camel bull can be sold for 100,000 Shillings [£770], whereas a big cattle bull can fetch 30,000 Shillings. There is one person from this village, who sold his camel bull in Moyale recently for 110,000 Shillings.^29^

Camels reproduce slowly but the demand for and price of camels has been rising (Mahmoud 2013). Live animals are sold at Moyale market and exported to Middle Eastern countries, where they have replaced camels formerly brought from Sudan and Somalia, sources which have been adversely impacted by insecurity (ibid).

Camels made other contributions to livelihoods. They were used to carry water, to collect firewood, and to plough, and they were hired out at rates of between 1500 and 3600 Shillings per day. Camels were rarely slaughtered for meat, but their milk was very important to household nutrition and income. Camels give more milk per day, and they give milk for a longer period than a cow (Dahl and Hjort 1976; Bollig 1992). Camel milk is highly nutritious, and considered locally to be especially good for people with conditions such as diabetes. There was a ready market for camel milk in Marsabit town, where it was enjoyed as a drink on its own, or in tea. Many camel herders interviewed had arrangements known as ‘*mabil’* through which they sold milk to individuals or businesses: the herders supplied camel milk daily, and were paid monthly. One Borana man had bought a motorbike with which he now ran a profitable *boda boda* (motorbike taxi) business, entirely from the profits from his camel milk: ‘I bought my *boda boda* with camel milk. Camel milk only,’ he said.^30^ Another man (a Garre) who had received a camel from a project commented ‘it is blissful to be back with camels,’^31^ and a Borana man who had sold cattle to buy camels explained:

If the whole world took to camels, there wouldn’t be any poverty… Until you own them, you don’t know the importance of camels. Until you learn the sweetness of the camels, you don’t know. But now I have learnt the sweetness.^32^

As the shift from cattle to camels has been partly driven by a desire to make use of new economic opportunities it could be argued that it does not represent a climate adaptation. But for the pastoralists in northern Kenya, the beauty of the camels is that they do the two things: they are resistant to drought and they are profitable. As one local livestock expert explained, ‘the camel is the animal for climate change and it is the animal for commerce’.^33^

The notion of ‘shifting’ from cattle to camels is appropriate as it is difficult to herd cattle and camels together: they move at different paces, they require different vegetation, and sometimes they attack each other (Dahl and Hjort 1976). But the shift was not always total: of the Borana who had bought camels, the majority still had cattle, although usually only a few. Of the ‘project’ beneficiaries, several Borana, Gabra and Garre also had a few cattle. The majority on the mountain from all groups was also involved in farming. The adoption of camels represented a form of diversification rather than a new initiative because farming and cattle had failed. True, farming has proved unreliable in recent years, and many cattle have died, especially in the 2009 and 2011 droughts, but not all were similarly adversely affected. On the Rendille side (to the south), many cattle were taken to areas around Maralal during the droughts. Many Borana took their cattle to the areas around Wajir, to Mega (in Ethiopia), to Elle Bor (near Forole), and to Chalbi. Although the Borana lost more cattle than the Rendille, if a household had sufficient resources to practise long-distance mobility, then a good portion of their livestock survived. For those who had bought camels at least, the diversification was not forced by a complete loss of cattle or agriculture-based livelihoods, but was a strategic innovation based on the perception that they are more profitable and more dependable in the current climate.

## Social, Cultural and Political Dimensions of Change

The data presented above suggest that the shift to camels has made households more resilient to drought and can also improve income and nutrition. Improved income builds resilience its literal sense, as a function of development (Conway 2009). But development depends on more than just income. In this section we examine the wider social, cultural and political ramifications of the shift to camels, each of which has the potential to strengthen resilience or to bring new risks that could undermine it.

Firstly, it is commonly held that camels are difficult to manage. Although this may be true when a male camel is rutting, in general, camels were regarded by many of our interviewees as having advantages in terms of the labour they required.^34^ Several women, for example, commented that the shift to camels had reduced their labour. As one Borana woman explained:

Camels have less work for women. Usually we cut grass for cattle calves. Camel calves can go with their mothers. They don’t need forage or watering. So the camel is less work.^35^

Some women herded camels when no one else was available, and women were responsible for taking camel milk to market. Although the labour burden of milk marketing was high, the women we interviewed valued their related control over this income. Finally, with the customary camel keepers, the Gabra and the Rendille, women did not milk, but with the new camel keepers, husbands and wives often milked together, and some interviewees saw this as a new form of cooperation:

Camel work is something that we do together. Camels can’t be managed by one person only, so … the man and woman have to work together.^36^

Secondly, pastoralists have often been viewed as conservative,^37^ and the cultural associations of Borana with cattle could be a reason why they might resist the adoption of camels, an identity marker of their ethnic neighbours. It has already been seen that most new camel keepers kept one or two cattle and some said that this was for ritual purposes: ‘It’s not like we have completely taken our feet out of cattle herding. For cultural purposes we keep some cattle.’^38^ Others were more reticent, commenting ‘You don’t just adapt to the camel, you have to adapt to the whole culture,’^39^ and ‘the elders do not like it.’^40^ But most went on to dismiss concerns about the cultural ramifications of the change, explaining that the new wealth acquired allowed a person to overcome any cultural difficulties. As one Borana man explained: ‘if you need cattle for bridewealth or burial, you can just sell your camels and buy cattle’.^41^

Finally, in terms of politics and inter-ethnic relations, we had hypothesized that the shift to camels would increase competition and potentially exacerbate conflict, which would certainly threaten any increase in resilience. However, our interviewees told us the opposite, stressing two points: in terms reminiscent of McCabe’s (2004) book on neighbouring Turkana, *Cattle Bring Us To Our Enemies*, interviewees stressed that camels were better than cattle in relation to conflict with ethnic neighbours, because of their character:

The disadvantage of the cattle is that arms follow cattle. There are more frequent cattle raids than camel raids. Cattle, because they always want pasture, they always lead you into other people’s territory. So you just follow them into conflict hotspots. If there is grass in those areas, you just have to go. But because camels can browse in one area for a long time, there is no risk of moving them around into any enemy’s land… The camel has more patience.^42^

In addition, the shift to camels did not trigger competition with lowland neighbours because the camel-husbandry practices on the mountain were different in several respects from those of the lowlands. On the mountain, the camels browsed in limited patterns of movement, and those in milk especially were kept close to the homestead. In times of drought, the camels were not taken further away from the mountain, where they might compete with others, but up to town, where they browsed on euphorbia fences that had been planted around urban compounds.

The succulent euphorbia provides the camels with nourishment *and* water. In the 2011 drought, it proved particularly important:

In the last drought, the animals that stayed around the mountain here survived... The camels were good. They never died of drought. Where you can call the camel area is the area on the mountain here… When there is euphorbia, the camel can stay without drinking water.^43^

Another Borana woman explained:

[During the drought] we followed the euphorbia all the way to town. When they [camels] feed on euphorbia they have more milk. It’s like rainy day milk.^44^

Owing to these practices of relying on urban euphorbia fences, the shift to camels did not trigger conflict between ethnic groups as might have been expected. In times of drought the camels provided nourishment for their owners, and for people in the growing town, literally by eating the town itself. In summary, the social, cultural and political ramifications of the shift to camels did not undermine the resilience they brought.

## Social Differentiation and Unexpected Risks

While the shift to camels around Marsabit Mountain has been welcomed, there are still some important questions about who can participate successfully in this new camel economy. Buying camels depended on access to significant financial capital. New camel keepers were paying anything from 20,000 Shillings to 100,000 Shillings (for a large, mature bull), depending on condition and availability. For some it was even difficult to raise the 7000 Shillings that was required to qualify for a ‘project’ camel. Two new female camel owners had received their camels as gifts from sons in formal employment; not everyone has the benefit of such resources or generosity.

Successful camel husbandry also required specialist knowledge that was obtained through contacts and experience. The man who had acquired his motorbike through selling camel milk had gained knowledge and opportunities through his position on a school camel committee. Additionally, this man was able to benefit from keeping his camels together with those of the school where they were herded by the school herder (for which he made a small financial contribution), leaving him free to run his new *boda boda* taxi business. His example arguably represents a development success, but the point remains that the opportunities he made use of were not available to everyone.

Other new camel owners learned skills in camel husbandry from Somali friends around Wajir or Moyale. Some received help from Gabra friends and relatives, although this was less common as relations between Borana and Gabra had become strained. As more Borana learned camel husbandry, help and advice became available from the more immediate community on the mountain, but continued to depend on an individual’s networks and social capital.

The differential ability to benefit from camels became particularly apparent in relation to the inputs that camels required. In the case of euphorbia fed to camels in times of drought, formerly, a compound’s fence could be browsed freely or in return for some milk; by 2012 it was increasingly sold.^45^ If a person lacked the resources to purchase euphorbia then the consequences could be severe. As one man explained:

Seven of my camels died in the [2011] drought. They were mature ones. They died because there was not enough forage. [I didn’t go to town] because the people charge for the euphorbia and I didn’t have the money.^46^

Similarly, the camels on the mountain required expensive medicinal inputs. The smaller Gabra and Rendille camels in the lowlands require few inputs (Hülsebusch and Kaufmann 2002). The water sources in lowlands are salty and contain important nutrients, and the camels are moved frequently to avoid ticks and other disease-carrying parasites. The ‘Somali’ camels on the mountain suffered from colds. Often camel enclosures were not moved and parasites thrived. Those whose camels were doing well were those who had the resources and the knowledge to purchase salt, insect repellents, de-wormers, vaccines, and medicines. One Rendille man estimated that he spent around 1000 Shillings a year on each of his camels, sometimes travelling to Meru (a round trip of more than 600 km) to purchase camel drugs. His knowledge and practices were exceptional, however. Most had little knowledge of camel diseases. Of the new camel owners who obtained a ‘project’ camel, only a minority had attended any kind of health training as part of receiving their camel.

Wider support for camel health was weak. Veterinary services were under-staffed and under-resourced. Community animal health workers, who at one time provided some animal health support, were no longer available. Veterinary specialists named rinderpest, trypanosomiasis, anthrax, haemorrhagic septicaemia, Rift Valley Fever, tuberculosis, brucellosis and camel pox, as all affecting camels in the area. Ticks, worms and colds also compromised camel health, and there was even talk of a new disease that had arrived in recent years. As one local camel expert explained:

Lots of things are not known about camel disease and camel health. Two diseases can wipe out an entire herd very quickly: trypanosomiasis and haemorrhagic septicaemia. There is also another disease and no one knows what it is, or even if it is bacterial or viral. It makes the glands on the neck swell and the nose becomes mucusy.^47^

Specialist camel drugs were hard to access. Camels were frequently given drugs designed for cows, sheep or goats. Research into ‘peri-urban camels’ in Isiolo County (south of Marsabit) also identified camel mortality as a serious problem, and veterinary services as inadequate (Noor *et al.* 2012; Shibia *et al.* 2013).

In short, camels improved resilience, but only for those who could afford to manage them well. If an individual had contacts, had knowledge, and could purchase appropriate medicines and euphorbia in times of drought, then their camels could support his/her livelihood and potentially lead to new forms of diversification, strengthening his/her livelihood in a virtuous circle. There was little wider support or any safety net to provide the inputs if an individual’s resources were lacking. As with other forms of diversification that have not led to improved livelihoods or to a greater ability to cope with risk (Little *et al.* 2001a), the diversification to camels has not necessarily improved the resilience of the poor.

In addition, new risks may also be associated with the new camel economy. Firstly, camels were well adapted to the drier conditions, but future scenarios also suggest that climate change may bring increased rain and flooding in the long run (Conway 2009). Under this scenario camels may do much less well.^48^ Secondly, poorly medicated camels may have brought increased health risks, especially when kept in close proximity to human settlements on the mountain: several of the diseases suffered by camels are zoonotic.^49^ For example, brucellosis and tuberculosis have negative impacts on human health that are known. But new and emergent camel diseases could bring much more widespread and devastating impacts, such as those being experienced elsewhere (for example, the new Middle East respiratory syndrome coronavirus (MERS-CoV) has killed 544 people up to 2015 and has been linked to camels).^50^

Thirdly, Marsabit residents were skeptical about some of the new camel husbandry practices described above. Herders were divided, for example, on the use of euphorbia. Most were enthusiastic about its benefits, but one or two questioned its long-term health impact on camels and on humans:

Now the camels look healthy and fat but probably the euphorbia might not be good for them. At the end of it all, the camels in the lowlands are better!^51^

Fourthly, the research has pointed to the ways in which this new ‘camel economy’ is a market-based and commodified economy. It makes use of the proliferating fenced compounds in urban and peri-urban settlements on the mountain, which have increased at the expense of common grazing land for cattle. Without romanticising the past, it is possible to claim that historically, if a herder experienced difficulties there were multiple forms of non-monetary, kin-based, clan-based and inter-ethnic support that they could draw upon (Schlee 1989; Little *et al.* 2008). Over recent decades, forms of inter-ethnic cooperation have declined (Schlee and Shongolo 2012), and the advantages brought by diversification to camels have not fully replaced those older sources of resilience. The new camel economy is an individualized, commodified, market-based economy that depends on the vagaries of the market for animals, meat and milk. As such, it is an adaptation strategy that has the potential to expose herders to new market-based risks (O'Brien and Leichenko 2000). The differential ability of individuals to participate in this new economy also suggests that, like neoliberal economies elsewhere, it may entrench higher levels of socioeconomic differentiation (Harvey 2007).

## Conclusions

The increasing preference for camels on higher land in northern Kenya can be understood as a climate adaptation strategy, as it is, in part at least, a response to experiences of lower rainfall and more frequent drought. The case challenges many portrayals of pastoralists in dryland areas of Africa as conservative and as having ‘low adaptive capacity.’ Here, adaptation strategies are ongoing, and it is the herders who are initiating change. The adaptation practices are relatively straightforward, are spontaneous and autonomous, and are being eagerly embraced. They show that adaptation strategies do not have to be costly and ‘painful’ (Adger *et al.* 2009a: 2). These camel-based adaptation strategies may also be sustainable (in the sense of being continued) as they are ‘owned by’ and not imposed on the people themselves.

The account also challenges portrayals of pastoralists as vulnerable to climate change shocks and stresses because of their isolation and lack of access to robust markets (Boko *et al.* 2007). The camel market in northern Kenya – so far – is alive and well, and the increase in camels is as much an adaptation to new economic conditions: to the rising price of camels; to the increasing demand for milk in the growing urban centres; and to the reduction of grassland pasture on the mountain and the increase in euphorbia fences. Some of these economic changes are also related to the changing political situation: at an international level, protracted conflicts elsewhere have created an opportunity for northern Kenya; at a local level, new Borana practices are linked to strained relations with the Gabra since the 1990s.

The increasing preference for camels can be said to have improved resilience in northern Kenya in its literal sense. As hardy, drought-resistant animals, camels provide the basis of livelihoods that are better able to cope with some of the unexpected and variable shocks and stresses that climate change may bring. Camels have been shown to contribute to household incomes and livelihoods (agriculture, firewood, nutrition), to lead to further forms of diversification, and some interviewees even claimed that camels could lead to more collaborative gender relations. But our research also identified four vital concerns and risks that require attention.

First, the above conclusions are true under a scenario in which the climate becomes warmer and drier, but climate change predictions also point to more rain and bouts of heavy flooding. Under the latter scenario, camels on the mountain may prove a more risky rather than a more resilient strategy.

Secondly, insufficient understanding of, and provision for, camel health means that there are high losses associated with camels and there may also be new camel-related risks to human health.

Thirdly, the market-based and commodified nature of the new camel economy means that there may be new risks attached to participation; in adapting to the vagaries of the climate, the new camel herders may become further exposed to the vagaries of the market (O’Brien and Leichenko 2000).

Fourthly, and relatedly, the case demonstrates the continuing significance of equity issues (Thomas and Twyman 2005; Nelson *et al.* 2007). In 2012, the switch to camels was a form of diversification that improved resilience for those who had resources; many questions have been raised about the extent to which it represented a successful form of resilience building for the poor, the most vulnerable to climate shocks.

These concerns and risks should not detract too much from the idea that camels can be the basis of a more resilient ‘adaptation pathway.’ On the contrary, they demonstrate that with appropriate and necessary support from government and development organizations for mobility, animal health, adequate nutrition and marketing, camels could improve resilience further and for more people. More detailed research is required to see whether or not these developments result in increasingly resilient livelihoods over time, but certain findings are already clear: the Borana interviewee’s assertion that ‘[i]f the whole world took to camels, there wouldn’t be any poverty’ is too simplistic. There are multiple risks and potential new inequalities linked to the shift to camels. The most important finding from our research, for state bodies and NGOs who would further the trend, is that wider support is needed for livestock keeping in general, and for camels in particular. It is not enough just to give an animal.

## Footnotes

1 Pacida, www.pacida.org/download/3.pdf, accessed 19 September 2013.

2 Pelling (2011) uses the terms ‘adaptation pathway’ to refer to different potential ‘ways of adapting’ that can be chosen by actors from a range of options.

3 1857 definition, Oxford English Dictionary, accessed December 4, 2015.

4 Commission of Revenue Allocation, Kenya County Fact Sheet 2011. https://www.opendata.go.ke/Counties/Kenya-County-Fact-Sheets-Dec-2011/zn6m-25cf (accessed 2 October 2013).

5 Figures from 2005 to 6 for Marsabit District from https://www.opendata.go.ke/Poverty/District-Poverty-Data-KIHBS-2005-6/pnvr-waq2? (accessed 12 November 2013).

6 Dahl and Hjort 1976; Rowley-Conwy 1988.

7 Gifford-Gonzalez and Hannotte 2013.

8 Halake Elema, interview 2012.

9 Qabale Boku, interview 2012.

10 Halake Elema, interview﻿ 2012.

11 The non-competitive nature of herding strategies, as well as the complementary reproduction patterns of camels and cattle, were also argued to be key to the alliance between the Rendille and Samburu (see Spencer 1973).

12 The first author since 1999; second author since 2008; and third author since 2005.

13 Dahl and Hjort discuss similar challenges, commenting, for example, that even ‘the act of counting domestic animals is often a cultural offence’ (1976; see also Sperling 1987; Adano and Witsenburg 2008).

14 Field, pers. comm. interview 2012.

15 This does not mean it did not have an influence.

16 Interview 2012.

17 Fifteen people self-described as Borana, two as Gabra Malbe, two Rendille and one Garre. If the Garre, Rendille and Gabra Malbe households had had camels in the past, they had not kept camels for more than three decades.

18 The Gabra and Rendille rarely sell their camels. As a consequence, even if new camel keepers preferred their camels (and they did not express a preference), they were not often available.

19 Interview 2012.

20 All currency calculations are approximates and based on the rate of £1 = 130 Shillings from September 2012.

21 Interview 2012.

22 At the time of our study, this distribution was in progress. On the mountain, most of the camels provided by projects were from the government or PISP projects.

23 Interview, Government Veterinary Services Officer, 2012’. He was visited in his office and asked, given his experience, to estimate the number of camels on the mountain. Other topics discussed included change in camel numbers, disease patterns, treatments and veterinary services provided.

24 Interview 2012.

25 As translated in Shongolo and Schlee 2007.

26 Molu Dabasso, interview 2012.

27 Bagaja Godana, interview 2012.

28 Borana borehole manager, interview 2012.

29 Jarso Golicha, interview 2012.

30 Hassan Malicha, interview 2012

31 Ario Mahmud, interview 2012.

32 Jarso Golicha, interview 2012

33 KARI employee, interview 2012.

34 All Borana interviewees considered milking to be the most labour intensive aspect of rearing camels. Individual households milked their camels differently, but the most productive milked once in the evening, once in the morning, and twice more in the night. For more on the labour requirements of camels, see Dahl and Hjort (1976), Schlee (1989), Sperling (1987) and Bollig (1992).

35 Guyatu Arero, interview 2012.

36 Barako Guyo, interview 2012.

37 For example, a recent report on northern Kenya claims ‘[o]n the whole, pastoralists are not innovative or willing to try new ideas’ (Watson and van Binsbergen 2008).

38 Man at Sagante wells, interview 2012.

39 Barako Guyo, interview 2012.

40 Jarso Golicha, interview 2012.

41 Jarso Golicha, interview 2012.

42 Jarso Golicha, interview 2012.

43 Malicha Robale, interview 2012.

44 Orge Galgallo, interview 2012.

45 The costs varied from 2000 to 5000 Shillings, depending on the amount of euphorbia.

46 Barako Guyo, interview 2012

47 KARI employee, interview 2012.

48 For an account of heavier losses in this area for camels, sheep and goats compared to cattle during the 1997/8 *el nino* floods see Little et al. 2001b.

49 They can pass between animals and humans.

50
https://www.gov.uk/government/uploads/system/uploads/attachment_data/file/461192/MERS-COV_RA_sep_2015_final.pdf (accessed 7 December 2015).

51 Tulu Roba, interview 2012

## References

[CR1] Adano WR, Witsenburg KM (2008). Pastoral Sedentarisation, natural resource management, and livelihood diversification in Marsabit District, northern Kenya (books 1 and 2).

[CR2] Adger WN, Huq S, Brown K, Conway D, Hulme M (2003). Adaptation to climate change in the developing world. Progress in Development Studies.

[CR3] Adger WN, Lorenzoni I, O'Brien K, Adger WN, Lorenzoni I, O'Brien K (2009). Adaptation now. Adapting to climate change: thresholds, values.

[CR4] Adger WN, Lorenzoni I, O'Brien K (2009). Adapting to climate change: thresholds, values, governance.

[CR5] Bernard HR (2011). Research methods in anthropology: qualitative and quantitative approaches.

[CR6] Boko, M., Niang, I., Nyong, A., Vogel, C., Githeko, A., Medany, M., Osman-Elasha, Tabo, R. and Yanda P. (2007). Africa. Climate change 2007: impacts, adaptation and vulnerability. In Parry, M.L., Canziani, O.F., Palutikof, J.P., van der Linden, P.J., Hanson, C.E. (eds.), Contribution of working group II to the fourth assessment report of the intergovernmental panel on climate change. Cambridge University Press, Cambridge, pp. 433–467.

[CR7] Bollig M (1992). East Pokot camel husbandry. Nomadic Peoples.

[CR8] Bollig M (2014). Resilience: analytical tool, bridging concept or development goal?. Zeitschrift für Ethnologie.

[CR9] Cannon T, Müller-Mahn D (2010). Vulnerability, resilience and development discourses in context of climate change. Natural Hazards.

[CR10] Catley A, Lind J, Scoones I (2013). Pastoralism and development in Africa: dynamic change at the margins.

[CR11] Conway, G. (2009). The science of climate change in africa: impacts and adaptation. Grantham Institute for Climate Change, Imperial College, Discussion Paper 1.

[CR12] Cordaid (2012). Managing resource variability in drylands: the role of pastoralists’ mobility in northern Kenya and southern Ethiopia.

[CR13] Dabasso B, Okomoli M (2015). Changing pattern of local rainfall: Analyis of a 50-year record in Central Marsabit, northern Kenya. Weather.

[CR14] Dahl G., and Hjort. (1976). Having herds: pastoral herd growth and household economy. University of Stockholm Social Anthropology, Stockholm.

[CR15] Field CR (2005). Where there is no development agency: a manual for pastoralists and their promoters.

[CR16] Fratkin E (1991). Surviving drought and development: Ariaal pastoralists of northern Kenya.

[CR17] Fratkin E, Roth EA (2005). As pastoralists settle: social, health and economic consequences of pastoral sedentarization in Marsabit District, Kenya.

[CR18] Gifford-Gonzalez D, Hannotte O, Lane PJ (2013). Domesticating animals in Africa. Mitchell, P.

[CR19] Hardin G (1968). The tragedy of the commons. Science.

[CR20] Harvey D (2007). Neoliberalism as creative destruction. The Annals of the American Academy of Political and Social Science.

[CR21] Hornborg A (2013). Revelations of resilience: from the ideological disarmament of disaster to the revolutionary implications of (p)anarchy. Resilience.

[CR22] Hülsebusch CG, Kaufmann BA (2002). Camel breeds and breeding in northern Kenya.

[CR23] Lamprey HF, Yusuf H (1981). Pastoralism and desert encroachment in northern Kenya. Ambio.

[CR24] Little PD, Smith K, Cellarius BA, Layne Coppock D, Barrett CB (2001). Avoiding disaster: diversification and risk management among east African herders. Development and Change.

[CR25] Little PD, Mahmoud H, Layne Coppock D (2001). When deserts flood: risk management and climatic processes among east African pastoralists. Climate Research.

[CR26] Little PD, McPeak J, Barrett CB, Kristjanson P (2008). Challenging orthodoxies: understanding poverty in pastoral areas of East Africa. Development and Change.

[CR27] Mahmoud HA, Catley A, Lind J, Scoones I (2013). Pastoralists' innovative responses to new camel export market opportunities on the Kenya/Ethiopia borderlands. Pastoralism and development in Africa.

[CR28] McCabe JT (2004). Cattle bring us to our enemies: Turkana ecology, politics, and raiding in a disequilibrium system.

[CR29] McCabe JT, Leslie PW, DeLuca L (2010). Adopting cultivation to remain pastoralists: the diversification of Maasai livelihoods in northern Tanzania. Human Ecology.

[CR30] Nassef M, Anderson S, Hesse C (2009). Pastoralism and climate change: enabling adaptive capacity.

[CR31] Nelson DR, Adger WN, Brown K (2007). Adaptation to environmental change: contributions of a resilience framework. Annual Review of Environment and Resources.

[CR32] Noor IM, Bebe BO, Guliye AY (2012). Analysis of an emerging peri-urban camel production in Isiolo County, northern Kenya. Journal of Camelid Science.

[CR33] O’Leary M, Brokensha D (1994). Patterns of range use, nomadism and sedentarization: the case of the Rendille and Gabra of northern Kenya. A river of blessings: essays in honor of Paul Baxter.

[CR34] Oba G (2013). Nomads in the shadows of empires: contests, conflicts and legacies on the southern Ethiopian-northern Kenyan frontier.

[CR35] O'Brien KL, Leichenko RM (2000). Double exposure: assessing the impacts of climate change within the context of economic globalization. Global Environmental Change.

[CR36] Pelling M (2011). Adaptation to climate change: from resilience to transformation.

[CR37] Robinson LW, Berkes F (2010). Applying resilience thinking to questions of policy for pastoralist systems: lessons from the Gabra of northern Kenya. Human Ecology.

[CR38] Rowley-Conwy P (1988). The camel in the Nile Valley: new radiocarbon accelerator (AMS) dates from Qasr Ibrim. Journal of Egyptian Archaeology.

[CR39] Schlee G (1989). Identities on the move: clanship and pastoralism in northern Kenya.

[CR40] Schlee G, Shongolo AA (2012). Pastoralism and politics in northern Kenya and southern Ethiopia.

[CR41] Shibia MG, Owuor G, Bebe BO (2013). Evaluation of losses of replacement heifers in pastoral and peri-urban camel herds in semi-arid northern Kenya. Pastoralism: Research, Policy and Practice.

[CR42] Shongolo AA, Schlee G (2007). Borana proverbs in their cultural context.

[CR43] Spencer P (1973). Nomads in alliance: Symbiosis and growth among the Rendille and Samburu of Kenya.

[CR44] Sperling L (1987). The adoption of camels by Samburu cattle herders. Nomadic Peoples.

[CR45] Stratford E, Bradshaw M, Hay I (2016). Qualitative research design and rigour. Qualitative research methods in human geography.

[CR46] The Royal Society (2009). Reaping the benefits: science and the sustainable intensification of global agriculture.

[CR47] Thomas DSG, Twyman C (2005). Equity and justice in climate change adaptation amongst natural-resource-dependent societies. Global Environmental Change.

[CR48] Thornton PK, van de Steeg J, Notenbaert A, Herrero M (2009). The impacts of climate change on livestock and livestock Systems in Developing Countries: a review of what We know and what We need to know. Agricultural Systems.

[CR49] Toni F, Holanda E (2008). The effects of land tenure on vulnerability to droughts in northeastern Brazil. Global Environmental Change.

[CR50] Toulmin C (2009). Climate change in Africa.

[CR51] Vincent K (2007). Uncertainty in adaptive capacity and the importance of scale. Global Environmental Change.

[CR52] Watson DJ, van Binsbergen J (2008). Livelihood diversification opportunities for pastoralists in Turkana, Kenya.

[CR53] Wilson T, Araya A, Melaku A (1990). The one-humped camel: an analytical and annotated bibliography 1980–1989.

